# Epithelial-to-mesenchymal transition in gallbladder cancer: from clinical evidence to cellular regulatory networks

**DOI:** 10.1038/cddiscovery.2017.69

**Published:** 2017-11-27

**Authors:** Sunwang Xu, Ming Zhan, Jian Wang

**Affiliations:** 1 Department of Biliary-Pancreatic Surgery, Renji Hospital, School of Medicine, Shanghai Jiao Tong University, Shanghai, China

## Abstract

Gallbladder cancer (GBC), with late diagnosis, rapid disease progression and early metastasis, is a highly aggressive malignant tumor found worldwide. Patients with GBC have poor survival, low curative resection rates and early recurrence. For such a lethal tumor, uncovering the mechanisms and exploring new strategies to prevent tumor progression and metastasis are critically important. Epithelial-to-mesenchymal transition (EMT) has a prominent role in the early steps of tumor progression and metastasis by initiating polarized epithelial cell transition into motile mesenchymal cells. Accumulating evidence suggests that EMT can be modulated by the cooperation of multiple mechanisms affecting common targets. Signaling pathways, transcriptional and post-transcriptional regulation and epigenetic alterations are involved in the stepwise EMT regulatory network in GBC. Loss of epithelial markers, acquisition of mesenchymal markers and dysregulation of EMT-inducing transcription factors (EMT-TFs) have been observed and are associated with the clinicopathology and prognosis of GBC patients. Therefore, EMT may be a detectable and predictable event for predicting GBC progression and metastasis in the clinic. In this review, we will provide an overview of EMT from the clinical evidence to cellular regulatory networks that have been studied thus far in clinical and basic GBC studies.

## Facts

- EMT markers and EMT-TFs are dysregulated in GBC tumor specimens.
- Multiple mechanisms are involved in EMT and thus regulate GBC tumor progression, including activation/inhibition of specific signaling pathways, transcriptional and post-transcriptional regulation and epigenetic alteration.
- Targeting EMT signaling pathways can be a potential therapeutic strategy for GBC treatment.

## Open questions

- Can targeting the EMT regulatory network be an effective strategy to achieve GBC growth prohibition or elimination?
- Is it possible to apply EMT markers as an immunohistochemical staining panel for GBC tumorigenesis or invasion ability validation in the clinic?
- How does EMT contribute to traditional chemoresistance in GBC?

Gallbladder cancer (GBC) is one of the most aggressive malignant tumors worldwide, and it represents 80–95% of biliary tract cancers (BTCs) based on autopsy studies and it ranks fifth among the most commonly occurring gastrointestinal cancers.^1,2^ The incidence of GBC is decreasing because of increased routine cholecystectomy; however, its mortality and prognosis have remained poor.^3^ The overall survival of GBC patients is only 6 months, with 5-year survival rates of 5–18%.^4,5^ This high mortality rate is attributable to the rapid progression of the disease and its highly aggressive behavior. Local invasion to the liver or adjacent organs, lymphatic metastasis, peritoneal dissemination and hematogenous metastasis are the main modes of malignant GBC development.^6^ Most patients with GBC are diagnosed at advanced or noncurative stages without surgical indication.^4^ Among patients who undergo curative resection, the recurrence rate remains high, and they typically present with distant recurrence with or without concomitant locoregional recurrence within 12 months after curative resection.^7,8^ For a tumor with this aggressive biological behavior and poor prognosis, it is critical to uncover the mechanisms of GBC progression and metastasis and identify potential therapeutic targets to improve clinical outcomes.

**Figure 1:**
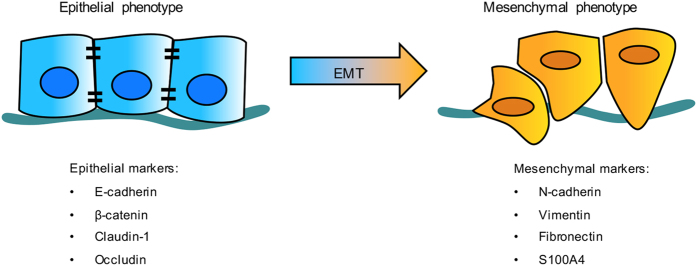
Cell marker changes in EMT. During EMT, epithelial cells lose their cell membrane epithelial markers and acquire mesenchymal markers and metastatic traits.

Over the past decades, epithelial-to-mesenchymal transition (EMT) has come to be regarded as a key process for tumor cells to acquire a more malignant phenotype. EMT is a reversible dynamic process that drives polarized epithelial cells to undergo multiple biochemical changes that allow them to gain a motile mesenchymal cell phenotype that loses cell–cell contacts and adhesion capacity.^9,10^ Mesenchymal cells can undergo a reverse process termed mesenchymal-to-epithelial transition to restore the epithelial phenotype. EMT contributes to embryonic development and tissue repair, but is also an early metastatic step for tumor cell invasion and migration, and it promotes tumor progression.^11^ Loss of major epithelial markers, such as E-cadherin, and overexpression of mesenchymal markers, including N-cadherin, Vimentin, Fibronectin and S100A4, often occur concomitantly during the EMT process (Figure 1).^10,12^ These dysregulated markers can be tested in tumor specimens via immunohistochemical staining at the protein level or with qPCR at the transcriptional level, and all are tightly associated with clinical parameters and survival.^13^ There are multiple EMT mechanisms involving different pathways and transcription factors, as well as epigenetic alterations, that either promote or suppress tumor development and progression.^14,15^ Through EMT, tumor cells acquire a mesenchymal phenotype and become capable of invading and migrating to local or distant regions, resulting in tumor progression and metastasis.

This review aims to summarize the clinicopathological and prognostic value of EMT markers in GBC patients and present an overview of the cellular regulatory EMT networks associated with GBC progression and metastasis that have been studied thus far. In addition, we provide a preview of current potentially effective chemical agents for targeting EMT.

## Clinical evidence of EMT in GBC

### Loss of epithelial markers

#### E-cadherin

E-cadherin, a subtype of the cadherin protein family, is encoded by the *CDH1* gene and is mainly expressed in epithelial cells. As a core component of adherens junctions, E-cadherin has a critical role in mediating and strengthening close membrane apposition between neighboring epithelial cells and participates in the overall polarization of epithelial cells.^16^ Dysregulated or delocalized E-cadherin expression is a hallmark of EMT and has a critical role in tumor cell progression and metastasis.^17^ E-cadherin is localized on the cell membrane of non-tumorous gallbladder epithelial cells, but E-cadherin downregulation or delocalization has been found in GBC tumorous sites via histopathology immunostaining, and a reduction in E-cadherin has been observed in 11.9–70.0% of GBC specimens (Table 1).^18–27^ The genomic instability of the *CDH1* gene, including microsatellite instability and loss of heterozygosity, was found to contribute to the reduced E-cadherin levels in GBC.^18^ Epigenetic silencing through promoter hypermethylation of the *CDH1* gene (11.1–40.9%) was also observed in GBC tissues, but its relevance in lowering E-cadherin expression is still unclear.^28,29^ In the transition from healthy gallbladder epithelia to inflammatory and tumorous epithelia, downregulated E-cadherin expression is consistently observed.^18,19^ In GBC, it has been confirmed that low E-cadherin expression is correlated with tumor progression and histological differentiation,^18,19,25–27^ pTMN stage and tumor grade,^23–27^ lymph node metastasis,^21,25,27^ and tumor size,^20^ and patients with lower E-cadherin expression exhibit poor survival outcomes.^20–22,26,27^

#### *β*-catenin

Another epithelial EMT marker is *β*-catenin, which is abundantly expressed on the cell membrane of non-tumorous specimens, but in tumorous GBC specimens *β*-catenin accumulates in the cytoplasm and/or nucleus.^30^ Decreased membranous *β*-catenin localization is correlated with tumor progression due to a loss of cell adhesive function.^19^ Increased cytoplasmic *β*-catenin accumulation and consequent translocation into the nucleus is often connected with a reduction in the E-cadherin level and altered expression of downstream genes, including genes in the Wnt signaling cascade and EMT-associated genes.^31,32^ However, the clinical value of the subcellular localization of the endogenous *β*-catenin in the cytoplasm and/or nucleus is still controversial. Kimura *et al.*^33^ and Ghosh *et al.*^34^ found that cytoplasmic and nuclear *β*-catenin accumulation were associated with poor histological differentiation grade and pT stage, but Choi *et al.* did not find any correlation between clinicopathological factors, overall survival or disease-free survival in GBC patients with cytoplasmic and nuclear *β*-catenin accumulation.^35^ Furthermore, Chang *et al.*^36^ found that cytoplasmic and nuclear *β*-catenin accumulation in GBC patients was correlated with less aggressive behavior, and especially cytoplasmic accumulation was associated with improved outcomes. Multiple serine/threonine residues encoded by the third exon of *β*-catenin can be phosphorylated by glycogen synthase kinase 3*β*, which leads to *β*-catenin degradation by the proteasome and prevents *β*-catenin cytoplasmic accumulation and nuclear translocation.^37^ Mutations in the *β*-catenin phosphorylation region can activate *β*-catenin signaling by upholding cellular *β*-catenin levels. However, *β*-catenin mutations, which were measured as activated mutations in exon three, were rare in GBC patients (ranging from absent to 9.1%) and were not correlated with its cellular accumulation.^36,38–40^

#### Claudin-1

Claudin-1 is a small transmembrane protein that maintains epithelial cell polarity and has a vital role in epithelium homeostasis.^41^ In GBC, membranous Claudin-1 expression was reduced in tumorous sites compared with that in healthy gallbladder epithelium and was associated with aggressive parameters and poor survival.^20,42^

#### Occludin

Occludin, which acts with Claudin-1 to form intercellular tight junctions on the cytoplasmic membrane, contributes to tight junction stabilization and optimal barrier function in the epithelium.^43^ Loss of Occludin led to failure of epithelial cell tight junctions and is associated with invasion and metastasis in GBC, but patients with higher Occludin expression in tumorous tissue survived longer than those with reduced or no Occludin expression.^20^

### Acquisition of mesenchymal markers

#### N-cadherin

A switch from E-cadherin to N-cadherin expression is a hallmark of EMT.^44^ N-cadherin is expressed by mesenchymal cells and is linked to motility and invasion in cancer. There is only one reported study that analyzed N-cadherin expression in GBC samples, and the study confirmed that increased N-cadherin expression was associated with advanced tumor stage, aggressive behavior and poor GBC patient survival.^45^

#### Vimentin

During EMT, Vimentin upregulation promotes the gain of functional and morphological mesenchymal cell characteristics in epithelial cells and drives the cellular architecture toward a migratory and invasive phenotype.^46^ The Vimentin expression level was increased in GBC specimens and is significantly higher in metastases than in primary tumors, which indicates that Vimentin is associated with GBC metastasis and lymph node metastases.^27,47^

#### Fibronectin

Fibronectin is a component of the extracellular matrix, which can be expressed and secreted by tumor cells, and excess Fibronectin creates a permissive environment for cancer cell growth and oncogenic progression.^48,49^ Fibronectin also acts as a mesenchymal marker for EMT. Cao *et al.*^50^ found that Fibronectin expression in GBC tissues was higher than that in the gallbladder epithelium in cholecystitis, and Fibronectin expression in stromal tissues was similar to the levels observed in GBC epithelia. Moreover, Fibronectin expression was significantly associated with histological grade, pT stage and poor survival in GBC patients.^50^

#### S100 calcium binding protein A4

S100 calcium binding protein A4 (S100A4) promotes EMT by inducing mesenchymal traits in tumors^51^ and is indicative of tumor progression and metastasis.^52^ As a mesenchymal EMT marker, excessive S100A4 expression was inversely correlated with the loss of E-cadherin in GBC^21^ and was associated with poor GBC patient survival.^26,53^ However, its clinical value with regard to clinicopathological factors is controversial. Chang *et al.* indicated that S100A4 expression was associated with aggressive GBC phenotypes,^26^ but Kohya *et al.* and Nakamura *et al.* found that there was no statistically significant correlation between S100A4 expression and clinicopathological factors in GBC.^21,53^

### Dysregulation of EMT-TFs

EMT is directly orchestrated by several transcription factors (EMT-inducing transcription factors (EMT-TFs)), including zinc-finger proteins of the SNAIL superfamily (Snail, Slug and Smuc), zinc finger and E-box-binding proteins of the ZEB family (ZEB1 and ZEB2) and the Twist family of bHLH transcription factors (Twist1 and Twist2).^54^ These EMT-TFs can suppress E-cadherin transcription by directly binding to the E-cadherin promoter and coordinate the inhibition of epithelial genes and activation of mesenchymal genes to initiate EMT and promote cancer progression.^55,56^

Snail is more highly expressed in GBC tissues than in paraneoplastic tissues, and increased Snail expression is associated with histological differentiation, aggressive traits (peritumoral tissue invasion and lymph node metastasis) and poor survival in GBC patients.^20^

ZEB1 expression was also increased in GBC, almost exclusively in the invasive sites but was rarely expressed in non-tumorous epithelia.^57^ Another EMT-TF, Twist1, was more strongly expressed in GBC tissues than in non-tumorous tissues, as measured by both immunohistochemistry staining and mRNA levels in specimens, and ectopic Twist1 expression was associated with shorter median survival rates, poor differentiation, local invasion and advanced TNM stage.^27^

## EMT regulatory networks in GBC

### Signaling pathways in EMT

#### Transforming growth factor-*β *signaling pathway

**Figure 2:**
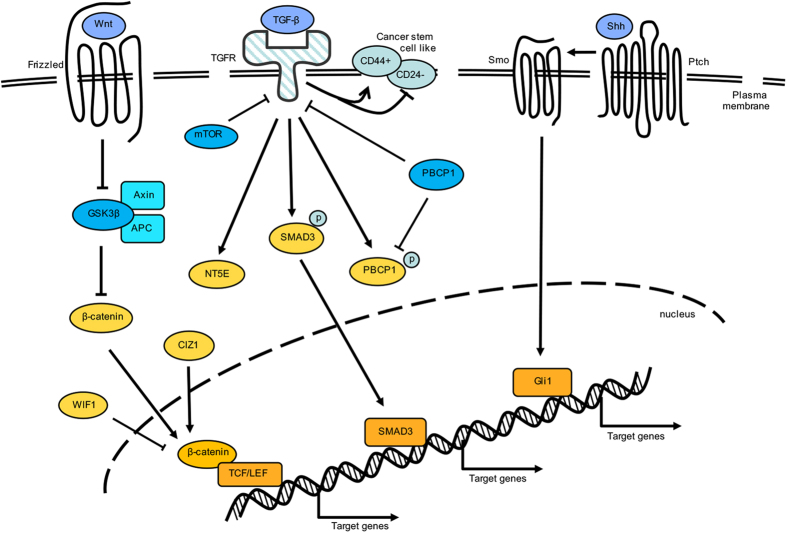
Multiple signaling pathways drive EMT in GBC cells. The TGF-*β* signaling pathway, Wnt signaling pathway and the Hh signaling pathway can govern the switch from an epithelial to a mesenchymal phenotype by upregulating oncogenic or decreasing tumor-suppressive gene expression. In addition, these signaling pathways can be activated or inhibited by multiple cellular factors.

Transforming growth factor-*β *(TGF-*β*) is one of the most well known and important EMT inducers (Figure 2).^58^ TGF-*β* expression is significantly increased in advanced-stage tumors compared with that in early-stage tumors, and it contributes to angiogenesis and macrophage infiltration in GBC.^59^ In advanced tumors, TGF-*β* promotes tumorigenesis and metastasis by inducing EMT via Smad-dependent and Smad-independent mechanisms.^60^ In GBC cell populations, the abundance of side population cells, which are known as cancer stem cells, was increased by TGF-*β*-induced EMT in a Smad3-dependent manner and reduced by withdrawing TGF-*β* or silencing Smad3 expression with siRNA.^61^ TGF-*β*-induced EMT in GBC cells was accompanied by phosphorylation of PCBP1 (poly r(C)-binding protein-1) at serine 43, but ectopic overexpression of PCBP1 attenuated the CD44^+^CD24^−^ stem-cell-like properties induced by TGF-*β*.^62^ With TGF-*β* treatment, expression of genes involved in the oxidation pathway, protein binding and adhesion in GBC cells were primarily altered.^63^ NT5E (etco-5′-nucleotidase, also called CD73), which was the most upregulated gene among 255 dysregulated genes after TGF-*β* treatment, suppressed E-cadherin expression and increased Vimentin expression to promote GBC proliferation and migration *in vitro*.^63^ TGF-*β*-dependent EMT can be inhibited with shRNA to downregulate mTOR levels, resulting in diminished invasion and migration ability in GBC.^64^

#### The Wnt signaling pathway

The Wnt signaling pathway is required for embryonic development and adult homeostasis, and deregulation of Wnt signaling has been implicated in developmental abnormalities and tumor progression.^65^ Activated Wnt signaling prevents *β*-catenin degradation by proteasomes followed by *β*-catenin nuclear accumulation and interaction with the TCF/LEF (T-cell factor/lymphoid enhancer factor) transcription factor to activate Wnt target gene translation.^31,65^ Sasaki *et al.* found that epithelial growth factor (EGF) enhanced EMT and stemness acquisition in GBC cells by activating Wnt signaling to induce *β*-catenin translocation into the nucleus and repressed the expression of E-cadherin.^58^ Cip7-interacting zinc-finger protein-1 physically interacts with TCF4 to activate *β*-catenin/TCF target gene expression, including c-Myc, Snail and Cyclin D1, to promote GBC cell growth and migration.^66^ In contrast, WIF-1 (Wnt inhibitory factor 1), an effective inhibitory factor of the Wnt signaling pathway, targeted *β*-catenin to inhibit tumor growth and induce apoptosis of GBC cells.^67^

#### The Hedgehog signaling pathway

The Hedgehog (Hh) signaling pathway can work alone or can engage in crosstalk with TGF-*β* and/or Wnt signaling pathways to initialize and maintain the EMT process.^68^ Three Hh ligands have been identified in the canonical Hh signaling pathway, Sonic Hh (Shh), Indian Hh (Ihh) and Desert Hh (Dhh). Hh signaling is orchestrated by Patched (Ptch) and Smoothened (Smo), which are both transmembrane receptors. Levels of Shh, its receptor Ptch and the downstream transcription factor Gli1 are frequently increased and significantly associated with tumor stage, lymph node metastasis, venous invasion, hepatic infiltration and poor GBC patient survival.^69^ Specifically, Gli1 was only detectable in the nucleus of tumorous gallbladder cells and was rarely observed in normal gallbladder cells.^70^ The treatment of recombinant human Shh can promote GBC cell proliferation and invasiveness *in vitro*, but silencing of Smo with siRNA can increase E-cadherin expression by downregulating matrix metalloproteinase (MMP)-3 and MMP-9 expression, resulting in E-cadherin accumulation on the cell membrane followed by a reduction in the percentage of spindle-shaped cells.^70^

### Transcription factors in EMT

#### Zinc-finger protein SNAI1 (Snail)

**Figure 3:**
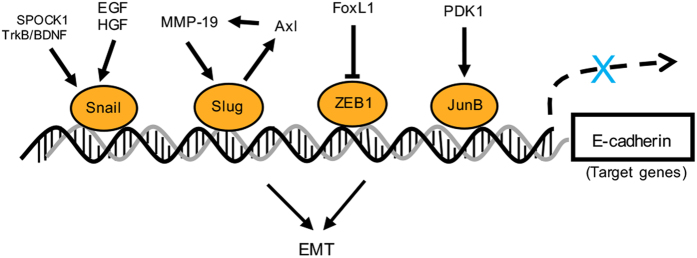
Transcription factor-mediated EMT regulation. EMT-TFs directly bind to their target gene promoter region to regulate target gene transcription and promote tumor cell migration, invasion and metastasis.

Snail is the central EMT regulator, and it has been observed to play a role in all EMT processes and is correlated with invasive behavior.^71^ Snail activates EMT by directly modulating epithelial and mesenchymal gene transcription (Figure 3). Snail represses E-cadherin expression by binding to E-box DNA sequences on the E-cadherin promoter region through its carboxy-terminal zinc-finger domain.^72^ Snail expression is controlled by both extracellular growth factors and intracellular networks. Snail has been observed to be activated by exogenous EGF and hepatocyte growth factor stimulation of GBC cells to induce EMT.^58^ Overexpression of SPOCK1 and TrkB/BDNF in GBC cells enhanced Snail expression and subsequently inhibited E-cadherin expression.^73,74^

#### Zinc-finger protein SNAI2 (Slug)

Slug, another member of the SNAIL zinc-finger protein superfamily, has a role similar to Snail in EMT progression by repressing E-cadherin expression and enhancing Vimentin and Fibronectin expression.^71^ Slug can be positively regulated by MMP-19 through binding to the Slug promoter region, and the subsequent Slug activation increased the expression of the receptor tyrosine kinase Axl to maintain Slug expression via a positive feedback loop and stabilize EMT in GBC cells.^75^

#### Zinc-finger E-box-binding homeobox 1

Zinc-finger E-box-binding homeobox 1 (ZEB1) is a transcription factor that drives EMT and cancer progression, and its expression often follows activation of Snail expression.^72,76^ ZEB1 was highly expressed in GBC-invasive sites and increased the GBC-invasive potential by repressing E-cadherin and T-cadherin expression and increasing N-cadherin and Vimentin expression at the transcriptional level.^57^ Furthermore, ZEB1 levels were reduced by Forkhead box L1 overexpression to rescue E-cadherin expression and inhibit GBC cell migration and invasion.^77^

#### JunB

The transcription factor activator protein-1 (AP-1) is one of the major effectors of gene transcription through binding to a consensus DNA sequence in the target gene promoter region.^78^ JunB belongs to the Jun subfamily of the AP-1 family. JunB exerts dual functions in tumors, acting as either an oncogene or tumor suppressor.^79^ However, JunB overexpression was confirmed in GBC tissues and was related to poor prognosis in GBC.^80^ JunB expression levels could be enhanced by 3-phosphoinositide-dependent protein kinase 1 to decrease E-cadherin expression and maintain the EMT phenotype.^80^

### Epigenetic alterations in EMT

#### Histone modification enzymes in EMT

Lysine-specific demethylase 1A (LSD1), also known as KDM1A (lysine-specific histone demethylase), was the first histone demethylase purified, and it can trigger H3K4me2 demethylation to H3K4me1 and H3K4me0.^81,82^ LSD1 can be recruited to the E-cadherin promoter by Snail to demethylate H3K4 in the E-cadherin promoter region, resulting in suppressed E-cadherin expression and enhanced cell invasion.^83^ LSD1 can cooperate with c-Myc to induce EMT, leading to GBC cell proliferation and invasion.^84^ LSD1 has been demonstrated to be upregulated in GBC tissues compared with paired normal tissues, and LSD1 upregulation indicates poor outcomes in GBC patients.^84^

Histone acetylation is involved in transcription activation, but deacetylation represses gene transcription. HDAC1, a member of the histone deacetylase (HDAC) family, catalyzes the removal of acetyl residues from histones assembled in inactive regions.^85^ During EMT, HDAC1 is recruited to the E-cadherin promoter by Snail to silence E-cadherin expression via deacetylation of histone 3 (H3) and histone 4.^86^ HDAC1 overexpression can cooperate with TCF-12 to promote EMT-TF (Snail, Slug and Twist2)-mediated transcription and subsequently inhibit E-cadherin transcription, which eventually leads to more invasive and metastatic traits in GBC cells.^87^

### Post-transcriptional regulation in EMT

#### MicroRNA

MicroRNAs (miRNAs) are short, highly conserved endogenous non-coding RNAs ~19–22 nucleotides in length that post-transcriptionally control gene expression by degrading mRNA or inhibiting protein translation through binding to the 3′ untranslated region (3′-UTR) of target genes.^88^ miRNAs negatively regulate numerous target genes involved in a variety of pivotal biological processes, such as cell growth, proliferation, differentiation and apoptosis. miRNAs also act as oncogenes or tumor suppressors during tumorigenesis.^89–91^ miRNAs target multiple components involved in epithelial integrity or mesenchymal traits to induce or suppress EMT and metastasis in cancer. In GBC, different sets of miRNAs can work as oncogenes (for example, miR-20a) to promote^92,93^ or tumor suppressors (such as miR-33a, miR-29c-5p, miR-101 and miR-122) to inhibit EMT by regulating their target gene expression (Table 2).^93–97^

miR-20a was induced by TGF-*β* in GBC cells and bound to the conserved 3′-UTR of Smad7 to negatively regulate its expression, and the downregulated Smad7 can transactivate miR-20a-enhanced *β*-catenin reporter gene activity, which resulted in *β*-catenin nuclear translocation, a subsequent reduction in E-cadherin expression and an increase in Vimentin expression.^92^ In contrast, miR-29c-5c, miR-101 and miR-122 negatively regulate their target genes, CPEB4, ZFX and PKM2, respectively, and can abrogate TGF-*β*-induced EMT and thus act as tumor suppressors to inhibit GBC cell proliferation, invasion and migration.^95–97^ In addition, miR-30a directly binds to Twist mRNA to suppress IL-6-induced EMT.^94^ All these oncogenic miRNAs and tumor-suppressive miRNAs are associated with GBC patient clinical outcomes.^92,94–96^

#### Long non-coding RNA

Long non-coding RNAs (lncRNAs) are a highly heterogeneous group of non-coding RNAs more than 200 nucleotides in length that lack protein-coding potential because of the absence of a discernable open reading frame. lncRNAs can be sorted into one or more of five broad categories: (1) sense, (2) antisense, (3) bidirectional, (4) intronic and (5) intergenic with respect to neighboring coding transcripts.^98^ lncRNAs act in cis or trans to regulate gene expression via multiple distinct molecular mechanisms, including transcriptional and post-transcriptional regulation, modulation of protein stability, acting as competing endogenous microRNA (ceRNA) sponges, modular scaffolding and chromatin remodeling.^99,100^ Emerging evidence has demonstrated that lncRNAs are dysregulated in various cancers and play essential roles in multiple cancer pathogenesis processes, partially by influencing the EMT process.^100–102^ Recently, there have been an increasing number of reports demonstrating that lncRNAs can promote aggressive GBC phenotypes by inducing EMT.^103^

When acting as ceRNAs, lncRNAs can complementarily bind to miRNA to suppress miRNA transcription and restore miRNA target gene expression and function. Thus far, there have been several studies showing that multiple lncRNAs are highly expressed in GBC tissues and activate oncogenes by negatively repressing miRNAs to induce EMT.^104–109^ For example, lncRNA Malat1 directly binds to miR-206 and restores KRAS and ANXA2 (annexin a2) expression, both of which are miR-206 target genes, forming a Malat1/miR-206/KRAS-ANXA2 axis to induce EMT via loss of E-cadherin and acquisition of Vimentin and Twist1 expression.^104^ Other lncRNAs, for example, lincRNA-00152,^105^ lncRNA-H19,^107^ lncRNA-TUG1^108^ and lncRNA-MINCR,^109^ can bind to miR-138, miR-194-5p, miR-300 and miR-26a-5p, respectively, and silencing lncRNA-H19 and lncRNA-TUG1 in GBC cells reversed TGF-*β*-induced EMT.^107,108^ These highly expressed oncogenic lncRNAs were also good indicators of GBC patient clinical outcomes and clinicopathological factors (Table 2). lncRNA-AFP1-AS1, lncRNA-ROR, lncRNA-KIAA0125 and linc-ITGB1 also act as oncogenic factors to decrease the expression of the epithelial markers E-cadherin or *β*-catenin and increase the expression of the mesenchymal markers Vimentin, Twist or Slug to induce EMT in GBC cells, but the underlying molecular mechanisms still needed to be clarified.^110–113^

#### Pseudogene

Pseudogenes were previously considered non-functional genomic relics without protein-coding ability.^114,115^ Recently, increasing evidence has demonstrated that pseudogenes are transcribed into non-coding RNAs that have multilayered biological functions in various cellular processes, especially in cancer.^116^ Several overexpressed pseudogene transcripts have been found to act as EMT inducers in GBC.^117,118^ The NmrA-like family domain containing 1 pseudogene Loc344887 can induce EMT in GBC cells by positively regulating Twist1 to promote GBC cell proliferation, migration and invasion.^117^ Downregulation of the PTEN mammalian homolog TPTE2 pseudogene, TPTE2P1, inhibited cell migration and invasion capacity by reducing EMT in GBC cells.^118^

## Targeted EMT therapeutics in GBC

To find potential therapeutic targets, it is critical to uncover the molecular basis and mechanistic properties of EMT. The above review summarized the dysregulated profiles in GBC based on clinical evidence and examination of the cellular regulatory networks associated with EMT. These studies provide potential therapeutic targets for GBC therapy. There are a few well-studied chemical agents that antagonize EMT in GBC (Table 3). Rapamycin is a pharmacological mTOR inhibitor that inhibits EMT by negatively regulating EMT-TFs. Rapamycin diminishes TGF-*β*-induced EMT by downregulating ZEB1/2 in a dose-dependent manner *in vitro* and decreases invasion and migration of GBC *in vivo.*^64^ Wachter *et al.* screened five potential anticancer drugs (DMAT, FH535, TBB, myricetin and quercetin) that targeted the Wnt signaling pathway in BTC cells, including three GBC cell lines.^119^ Among them, FH535, DMAT and TBB showed a high cytotoxic effect in all cell lines through inhibition of the Wnt signaling pathway and apoptosis induction. Decitabine (5-aza-2-deoxycytidine) might be a candidate drug to eliminate genomic hypermethylation to restore WIF-1 expression levels by inhibiting the Wnt signaling pathway in GBC.^67^ Cyclopamine, a specific Hh signaling pathway antagonist of Smo that was used for targeting high Smo-expressing tumors, could potentially be an effective inhibitor to suppress GBC cell proliferation and invasion *in vitro*.^70^ The HDAC inhibitor suberoylanilide hydroxamic acid (SAHA) increased E-cadherin expression and inhibited GBC cell growth by targeting HDAC1/2.^120^ Furthermore, the anticancer ability of SAHA was reinforced by silencing EZH2 with siRNA, which indicated that combined therapy with an HDAC inhibitor and an EZH2 inhibitor might be used to inhibit GBC proliferation. Recently, it was found that PIK3CA E545K mutation promoted GBC progression via binding to EGFR followed by downstream Akt activation and EMT promotion. After treatment with the PI3K p110α-selective inhibitor A66, the proliferation rate of GBC cells was significantly reduced both *in vitr*o and *in vivo*.^121^

## Future perspectives

Thus far, there is no doubt that EMT has a pivotal role in GBC development. Multiple studies have revealed the EMT markers and EMT-TFs that are closely associated with GBC progression, metastasis and prognosis in the clinic, which indicates that EMT can be a detectable and predictable event in GBC progression and metastasis in the clinic. In addition, signaling pathways, transcriptional and post-transcriptional regulation and epigenetic alterations have already been shown to form a comprehensive EMT regulatory network in GBC cells. However, knowledge of other mechanisms, such as the tumor microenvironment or tumor immunology, related to EMT in GBC is still lacking.

An effort to uncover the molecular mechanisms involved in tumor development is essential for us to find effective therapeutic targets to improve clinical outcomes, because the connection between regulatory networks and EMT events provides potentially favorable therapeutic targets for inhibiting GBC progression and metastasis. Further understanding of how to target these EMT processes by applying EMT inhibitors alone or in combination with other drugs is necessary for improving GBC therapeutic strategies.

## Additional information

**Publisher’s note:** Springer Nature remains neutral with regard to jurisdictional claims in published maps and institutional affiliations.

## Figures and Tables

**Table 1:** Clinical relevance of EMT in human GBC

	*Number of samples*	*Expression/localization*	*% of cases*	*Association with clinical parameters*	*Ref.*
*Epithelial markers*					
E-cadherin	36	Down	66.7	Tumor progress	^18^
	10	Down cytoplasmic	30.0 80.0	Tumor progress	^19^
	108	Down	46.3	Poorer survival Tumor size Lymph node metastasis Peritumoral tissue invasion	^20^
	37	Down	62.2	Poorer survival Lymph node metastasis	^21^
	49	Down	55.1	Poorer survival	^22^
	49	Down	65.3	pT category	^23^
	73	Delocalization	50.7	pTNM stage	^24^
	46	Negative	11.9	Histologic differentiation Clinical stage Lymph node metastasis	^25^
	132	Down	27.3	Poorer survival Histology pM category Tumor grade	^26^
	20	Down (mRNA)	70.0	Shorter median survival rates Differentiation degree Local invasion Lymph node metastasis TNM stage	^27^
* β*-catenin	10	Down Nuclear	50.0 50.0	Tumor progress	^19^
	49	Down Cytoplasmic Nuclear	79.6 40.8 12.2	Unknown	^22^
	21	Cytoplasmic Nuclear	47.6 47.6	Poorer histological differentiation grade	^33^
	80	Nuclear	75.0	pT category Tumor differentiation	^34^
	68	Negative Cytoplasmic Nuclear	11.8 45.6 22.1	Negative: low TNM stage Cytoplasmic/nuclear: unknown	^35^
Claudin-1	108	Down	54.6	Poorer survival Tumor size Lymph node metastasis Peritumoral tissue invasion	^20^
	34	Down	Unknown	Unknown	^42^
Occludin	108	Down	51.9	Poorer average survival Tumor size Lymph node metastasis Peritumoral tissue invasion	^20^
*Mesenchymal markers*					
N-cadherin	80	Up	55.0	Poorer survival Differentiation Tumor size TNM stage Lymph node metastasis Invasion	^45^
Vimentin	20	Up (mRNA)	70.0	Lymph node metastasis	^27^
	6	Up	—	Promote metastasis	^47^
Fibronectin	116	Positive/membrane	64.7	Histologic grade pT stage Poorer survival	^50^
S100A4	37	Up	62.2	Not correct with clinicopathologic factors but inverse correlation with E-cadherin expression	^21^
	120	Up	10.8	Poorer survival Venous invasion pM stage Tumor stage	^26^
	60	Positive	41.6	Poorer survival But not correct with histologic grade and TNM stage	^53^
*EMT-inducing transcription factors (EMT-TFs)*					
Snail	108	Up	57.4	Poorer survival Adenocarcinoma differentiation Lymph node metastasis Peritumoral tissue invasion	^20^
ZEB1	30	Up	76.7	Unknown	^57^
Twist1	20	Up (mRNA)	70.0	Shorter median survival rates Differentiation degree Local invasion Lymph node metastasis TNM stage	^27^

**Table 2:** Non-coding RNAs associated with EMT in GBC

	*Property*	*EMT markers*	*Target genes/pathway*	*Aggressive traits*	*Expression in GBC*	*Association with clinical parameters*	*Ref*
*miRNA*							
miR-20a	Oncogenic	↓E-cadherin↑Vimentin	Smad7/*β*-catenin axis	↑Migration↑Invasion	Up	Poorer overall survival, tumor size, local invasion, distant metastasis, TNM stage	^92^
miR-33a	Tumor suppressor	↑E-cadherin↓Vimentin	Twist	↓Migration↓Invasion↓Tumor growth	Down	Poorer metastasis free and overall survival, clinical stage, lymph node metastasis	^94^
miR-29c-5p	Tumor suppressor	↑E-cadherin↑*β*-catenin↓Vimentin	CPEB4/MAPK pathway	↓Proliferation↓Migration↓Invasion	Down	Poorer overall survival, poorer disease-free survival, lymph node metastasis	^95^
miR-101	Tumor suppressor	↑E-cadherin↑*β*-catenin↓Vimentin	ZFX/ MAPK-ERK‘Sand pathway	↓Proliferation↓Migration↓Invasion	Down	Poorer overall survival, tumor size, tumor invasion, lymph node metastasis, TNM stage	^96^
miR-122	Tumor suppressor	↑E-cadherin↓Vimentin	PKM2	↓Proliferation↓Migration↓Invasion	Down	Unknown	^97^
							
*lncRNA*							
Malat1	Oncogenic	↓E-cadherin↑Vimentin↑Twist	miR-206/ANXA2, KRAS	↑Proliferation↑Invasion↑Apoptosis	Up	Poorer overall survival, tumor size, lymph node metastasis	^104^
lincRNA-00152	Oncogenic	↓E-cadherin↑Vimentin	miR-138/HIF-1α	↑Migration↑Invasion	Up	Poorer overall survival, pT status, pN status	^105^
lncRNA-H19	Oncogenic	↓E-cadherin↑Vimentin↑Twist	miR-194-5p/AKT2	↑Proliferation↑Invasion	Up	Poorer overall survival, tumor size, lymph node metastasis	^106,107^
lncRNA-TUG1	Oncogenic	↓E-cadherin↑Vimentin	miR-300	↑Proliferation↑Invasion	Up	Lymph node metastasis	^108^
lncRNA-MINCR	Oncogenic	↓E-cadherin↑Vimentin	miR-26a-5p/EZH2	↑Proliferation↑Invasion↓Apoptosis	Up	Poorer overall survival, tumor volume, lymph node metastasis	^109^
AFAP1-AS1	Oncogenic	↓E-cadherin↑Vimentin↑Twist	Unknown	↑Proliferation↑Invasion	Up	Poorer survival, tumor size	^110^
lncRNA-ROR	Oncogenic	↓E-cadherin↑Vimentin↑Twist	Unknown	↑Proliferation↑Migration↑Invasion	Up	Poorer survival, tumor size, lymph node metastasis	^111^
KIAA0125	Oncogenic	↓*β*-catenin↑Vimentin	Unknown	↑Migration↑Invasion	Unknown	Unknown	^112^
Linc-ITGB1	Oncogenic	↓*β*-catenin↑Vimentin↑Slug	Unknown	↑Proliferation↑Migration↑Invasion	Unknown	Unknown	^113^
							
*Pseudogene*							
Loc344887	Oncogenic	↓E-cadherin↑N-cadherin↑Vimentin↑Twist	Unknown	↑Proliferation↑Migration↑Invasion	Up	Tumor size	^117^
TPTE2P1	Oncogenic	↓*β*-catenin	Unknown	↑Migration			
				↑Invasion	Unknown	Unknown	^118^

↑, upregulated or enhanced; ↓, downregulated or inhibited.

**Table 3:** Effects of small-molecule inhibitor on EMT in GBC

*Inhibitors*	*Targets*	*Effects*	*Mechanisms*	*Cells*	*Ref.*
Rapamycin	mTOR	↓Invasion ↓Migration	↓mTOR effectors ↓ZEB1/2 ↓Vimentin, ↑E-cadherin	GBC-SD	^64^
Cyclopamine	Smo	↓Proliferation ↓Invasion	Unknown	GBd15 TGBC2TKB	^70^
FH535, DMAT, TBB	Wnt	↓Viability ↑Apoptosis	↓Wnt effectors ↓Cyclin D1 ↑Caspase 3/7 ↑p27	MzChA-1 MzChA-2 GBC	^119^
Decitabine	DNA methyltransferase	Unknown	↓DNA methylation ↑WIF-1	GBC-SD	^67^
SAHA	HDAC	↓Cell growth	↓HDAC1/2, EZH2 ↑E-cadherin ↑p21	TGBC2TKB	^120^
A66	PI3K p100α	↓Proliferation	↓PI3K-AKT effectors	GBC-SD NOZ	^121^

↑, upregulated or enhanced; ↓, downregulated or inhibited.
